# The biological functions of proanthocyanidin and its application in pig production

**DOI:** 10.3389/fvets.2025.1565501

**Published:** 2025-03-12

**Authors:** Aiying Yu, Lingli Sun, Lian Chen, Dan Wang, Zhiyi Li, Yuan Wang, Zhenjiang Wang, Sen Lin

**Affiliations:** ^1^Key Laboratory of Urban Agriculture in South China, Sericultural and Agri-Food Research Institute, Guangdong Academy of Agricultural Sciences, Guangzhou, China; ^2^Tea Research Institute, Guangdong Academy of Agricultural Sciences, Guangzhou, China; ^3^Guangdong Provincial Key Laboratory of Tea Plant Resources Innovation and Utilization, Guangzhou, China

**Keywords:** proanthocyanidins, biological functions, antioxidant, anti-inflammatory, pigs

## Abstract

Proanthocyanidins (PACs) are natural polyphenolic compounds widely distributed in various plants, which are mixtures of oligomers and polymers formed by the polymerization of different numbers of catechins and epicatechins. PACs exhibit a range of biological activities, including antioxidant, anti-inflammatory, anti-cancer, anti-atherosclerotic, hypoglycemic, and antihypertensive effects, as well as the ability to regulate intestinal flora and promote fat metabolism. These properties render PACs highly promising for applications in the food, pharmaceutical, and cosmetic industries, garnering substantial interest from researchers globally. Additionally, PACs demonstrate significant nutritional benefits in animal husbandry. Dietary PACs can enhance animal growth, mitigate oxidative stress, decrease feeding expenses, and offer an environmentally friendly, antibiotic-free alternative. Therefore, PACs have great application potential in the field of pig production. This article reviews the basic properties, biological functions, and research status and application in pig production of PACs, aiming to provide theoretical guidance for the development of substitute antibiotic feed additives.

## Introduction

The wide use of antibiotics as feed additives has lasted for decades because of its beneficial effects in farm animals. However, the misuse of antibiotics has led to the emergence of multi-drug resistant bacteria, including vancomycin-resistant Enterococcus, Methicillin-resistant *Staphylococcus aureus*, and carbapenem-resistant Enterobacteriaceae, posing a significant threat to both animal and human health (1, 2). To cope with this, the Ministry of Agriculture and Rural Affairs of China issued a ban on antibiotics in feed in 2020. Since then, the feed and livestock industry has been highly concerned with the development and research of new types of feed additives that can replace antibiotics, which has become key to the sustainable development of the livestock industry of China. Plant extracts, acidifiers, antimicrobial peptides, probiotics, and enzyme preparations are considered to have good potential as alternatives to antibiotics. Notably, plant extracts as feed additives offer numerous advantages in the livestock industry. They are derived from natural sources, making them environmentally friendly and reducing the need for synthetic chemicals. Numerous studies have shown that plant extracts including tannins, saponins, flavonoids and important oils can enhance nutrient absorption, increase stress resistance ability, boost immune systems, and improve the quality and taste of meat products (2–7).

Polyphenols, derived from vegetables and fruits, have garnered significant research interest due to their health-promoting effects. Proanthocyanidins (PACs) are the second most abundant natural phenolic substances in nature after lignin (8), which exhibit multiple biological activities, including anti-inflammatory, antioxidant, lipid-regulating, gut-protective, and anticancer effects (9–12). It have been proven that the antioxidant effect of PACs is stronger than other antioxidants, such as Vitamin E (VE), Vitamin C (VC), and *β*-carotene, and they can be rapidly absorbed by the body (13, 14). The role of dietary procyanidins as health-protective agents has gained significant attention in nutritional research (15, 16). Epidemiological studies have demonstrated that regular consumption of procyanidin-rich foods is associated with reduced incidence of inflammatory conditions and multifactorial diseases, including metabolic syndrome, atherosclerosis, and cancer (17–19). Research in recent years have demonstrated that dietary supplementation of PACs can promote growth, minimize inflammation and oxidative stresses, modulate gut microbial environment and improve quality of animal products in pigs, poultry and other farm animals (20–24).

Widely distributed in various plants, PACs have been extracted from different agricultural by-products including grape seed, lotus seed, *Litchi chinensis* pericarp, barley bran and so on (25–28). Numerous studies have demonstrated that the reuse of agricultural by-products not only reduces costs but also enhances environmental conditions. Given their beneficial effects and the abundance of sources, the extraction of PACs from agricultural by-products and their subsequent use as feed additives offer a promising approach to seeking antibiotic alternatives. This review aims to elucidate the structure, sources, extraction methods, absorption, metabolism, and biological functions of PACs. It also seeks to summarize the effects of their application in pig production, thereby assessing their potential as feed additives for pigs.

## The basic characteristics of PACs

### The chemical structure and origin of PACs

**Figure 1:**
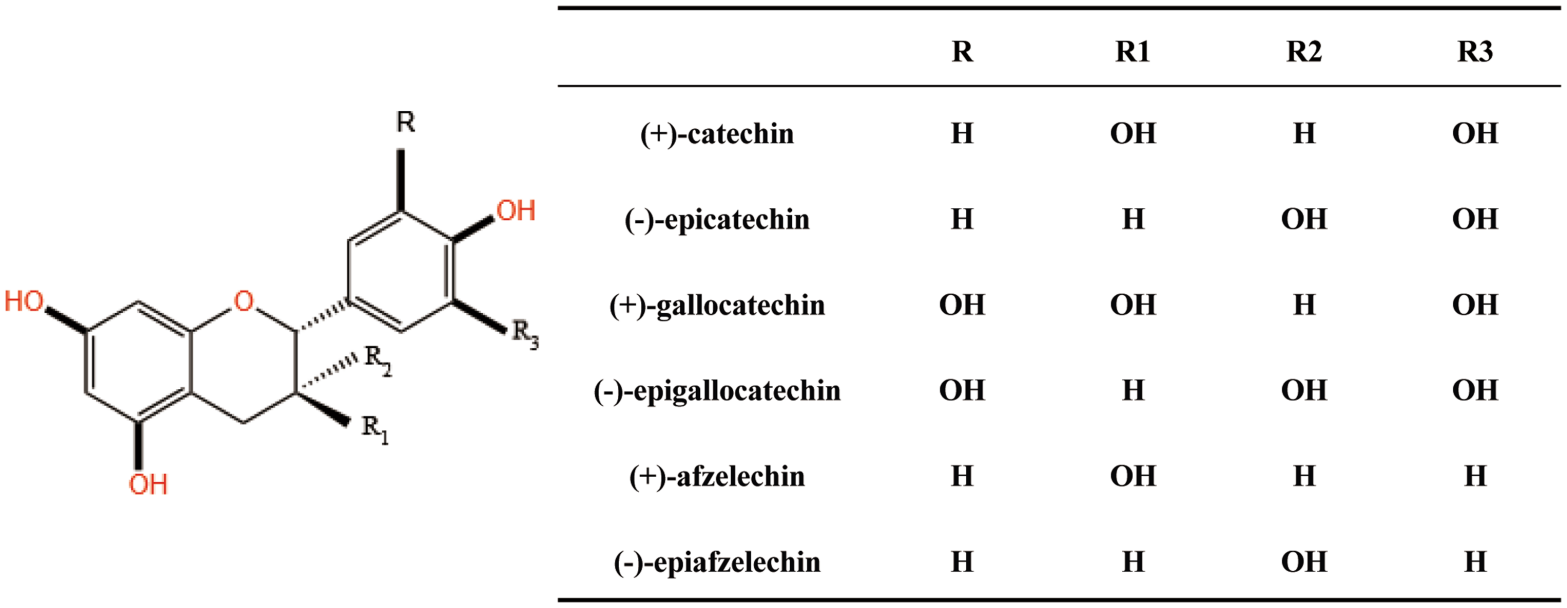
The basic structural unit of proanthocyanidins.

PACs also known as condensed tannins can be transformed into anthocyanins through degradation and oxidation when subjected to heat in an acidic medium which is the reason for their designation as proanthocyanidins (29). PACs are flavonoid polymers formed by the condensation of various amounts of catechins or epicatechins through C-C bonds with the basic structural unit being flavan-3-ol (Figure 1). Flavan-3-ol is a class of important flavonoid compounds containing two benzene rings (A B) connected by a heterocyclic ring (C) which is an enone pyran or pyrone. They exhibit different structures depending on whether the hydroxyl group is substituted whether the C3 hydroxyl position is gallated and the different chirality carbon atom configurations. Among these structures the B ring with two hydroxyl groups forms catechin (CAT) an isomer of epicatechin (EC). Based on the way the monomers are connected the dimeric PACs can be divided into A and B types. PAC-A is connected by both C-C and C-O-C bonds. PAC-B is combined through a single C-C bond between monomers (C4-C6 or C4-C8 flavan bond). PAC-A is more stable than PAC-B due to the additional C2-O7 covalent bond (30). PAC-A and PAC-B can interconvert under certain conditions (31). Their different structures also lead to differences in biological activity. Most plants contain only PAC-B while PAC-A is detected in only a few specific plants such as cranberries peanuts persimmon skins etc. (32, 33). Thus PAC-B is the predominant type of PAC in nature. According to the degree of polymerization (DP) PACs can be divided into oligomeric proanthocyanidins (OPC) and polymeric proanthocyanidins (PPC). Components with DP less than 5 are called OPC and those with DP greater than 5 are called PPC. The DP of PACs varies greatly due to different sources so the mean degree of polymerization (mDP) is a commonly used method to measure the average number of monomer subunits. The degree of polymerization of PACs also affects their biological activity and the neuroprotective effect is positively correlated with the degree of polymerization (34).

PACs are widely present in plants in nature with high contents found in the fruits of cocoa (35), persimmon (32), litchi (36), myrica (37), strawberry (38), peanut (33), and other plants. High levels of PACs have also been extracted and detected in some food by-products such as grape seeds hawthorn stone fruit peel litchi fruit peel Xinyang Maojian green tea cinnamon bark Platycodon stem and dandelion leaves (39). In addition PACs are also present in beverages processed from plant materials such as wine red wine and fruit juice (40–42). The total content average degree of polymerization and structural types of PACs vary greatly among different plants and even at different stages of the same plant (39, 43)

### Extraction, separation and purification of PACs

As technology advances, the optimization of proanthocyanidins (PACs) extraction methods is ongoing. The selection of extraction techniques and protocols is contingent upon the distinct attributes of raw materials, PACs types, and their polymerization levels. The extraction methods for proanthocyanidins mainly include organic solvent extraction, ultrasonic-assisted extraction, supercritical CO2 extraction, microwave-assisted extraction, and enzymatic extraction.

Similar to other polyphenolic compounds, proanthocyanidins are commonly extracted using solvent-based methods. The choice of solvent significantly influences the extraction efficiency, with acetone-water mixtures typically outperforming ethanol-water solutions. To enhance stability and facilitate the release of cell wall-bound proanthocyanidins, the extraction solvent can be adjusted to a pH of 4–6 with the addition of acetic acid, which also enhances extraction efficiency (44). Factors such as extraction duration, temperature, and auxiliary treatments notably affect the efficiency. Khanal et al. (45) found that soaking raw materials overnight in the extraction solvent prior to the process increased the proanthocyanidin yield by 24–100% compared to traditional extraction methods. Beyond conventional solvent extraction, various auxiliary techniques, including ultrasonic-assisted extraction, microwave-assisted extraction, and enzyme-assisted extraction, have been effectively applied (46–48). These physical and chemical methods are designed to disrupt the plant cell wall, enhancing solvent accessibility to proanthocyanidin molecules and thereby improving overall extraction efficiency.

The prevalent method for purifying proanthocyanidins remains column chromatography, which is particularly effective for the separation of highly polymerized proanthocyanidins. Gu et al. (49) employed a Sephadex LH-20 column and a 70% acetone-water mixture as the eluent to purify proanthocyanidins from blueberries. Sui et al. (50) utilized a medium-pressure chromatography column filled with Toyopearl HW-40S to isolate proanthocyanidin oligomers from lychee fruit shells. Proanthocyanidins, rich in hydroxyl groups, are readily adsorbed by polyamide, leading some researchers to use polyamide-packed solid-phase extraction columns for their purification, followed by elution with an 85% ethanol-water solution. The main disadvantage of this method is that the high boiling point of N,N-dimethylformamide makes the eluate difficult to concentrate. High-speed counter-current chromatography (HSCCC), a novel separation technique that capitalizes on the distribution equilibrium of samples between two immiscible solvents, has gained broad application in the purification of natural products. This method’s lack of irreversible adsorption between the sample and packing material results in a high recovery rate and enables the high-purity separation of complex mixtures. Zhang et al. applied HSCCC to separate proanthocyanidins from grape seeds, yielding seven fractions with a degree of polymerization ranging from 1.44 to 6.95 (51). By integrating HSCCC with gel column chromatography or preparative liquid chromatography, they achieved 17 proanthocyanidin compounds with purities between 70.0 and 95.7%. Li et al. successfully separated proanthocyanidins from cocoa beans using a solvent system comprising n-hexane, ethyl acetate, and water in a 1:50:50 ratio, obtaining compounds with varying degrees of polymerization.

### The absorption and metabolism characteristics of PACs

Since ancient times, traditional Chinese herbal medicines, which are rich in proanthocyanidins (PACs), have been utilized for the prevention and treatment of a variety of diseases. Extensive mouse experiments have demonstrated that PACs do not exhibit mutagenicity in either somatic or germ cells, and no adverse effects were observed in blood routine, blood biochemistry, and organ index assessments. Acute toxicity tests have likewise shown no toxic symptoms across all dosage groups. Studies involving piglets, broiler chickens, and mice have further confirmed that PACs pose no detrimental impact on animal health, establishing their non-toxic and safe profile suitable for broad application in diverse food products.

The bioavailability of PACs is influenced by their molecular structure, DP, interactions with other dietary components, the activity of gastrointestinal enzymes, and the gut microbiota composition. PACs exhibit robust acid resistance, remaining unabsorbed in the stomach, where they undergo partial degradation and interact with the food matrix. In the small intestine, some PACs are broken down into oligomers, while monomers of PACs, specifically A1, A2, and B2 type OPCs, are absorbed by the intestinal mucosa at a comparatively low rate (52). The portion of OPC and PPC that is not absorbed by the gastrointestinal tract is metabolized by gut microorganisms into various metabolites such as phenolactone and phenolic acids, which are then absorbed and circulated throughout the body to exert biological effects (52). The primary metabolites of PACs found in plasma include monomers, dimers, and their methylated and glucuronidated derivatives (53). PACs metabolites are even detectable in saliva (54). The unabsorbed PACs and their metabolites are ultimately excreted from the body via feces.

## Biological functions of PACs

### Antioxidant and anti-inflammatory effects

The hydroxyl groups present in the structure of PACs provide numerous hydrogen-donating sites, enabling them to interact with reactive oxygen species (ROS) and free radicals. This interaction effectively halts the chain reaction of free radicals, with PACs demonstrating a significantly greater capacity for free radical scavenging compared to vitamins C and E. They are thus considered potent scavengers of oxygen-free radicals and inhibitors of lipid peroxidation. Additionally, PACs shield tissues from oxidative stress-induced damage by neutralizing ROS or through the chelation of metal ions (55). The capacity of PACs to neutralize ROS is intimately linked to their DP. Studies have indicated that when PACs possess a polymerization degree of 3, their ROS clearance activity is augmented. However, an increase beyond this degree of polymerization can diminish the ROS clearance activity. PACs are capable of significantly slowing the degradation of nuclear factor-erythroid 2-related factor 2 (Nrf2) and markedly promoting its translocation into the nucleus. This, in turn, upregulates the expression of antioxidant enzymes and bolsters the body’s antioxidant defenses (56). Furthermore, the regulation of protein expression related to mitochondrial autophagy can be achieved by upregulating the expression of p- C-Jun N-terminal kinase (JNK)/Forkhead transcription factor (FOXO)3a, downregulating ROS levels, and enhancing the expression of antioxidant proteins (57).

**Figure 2:**
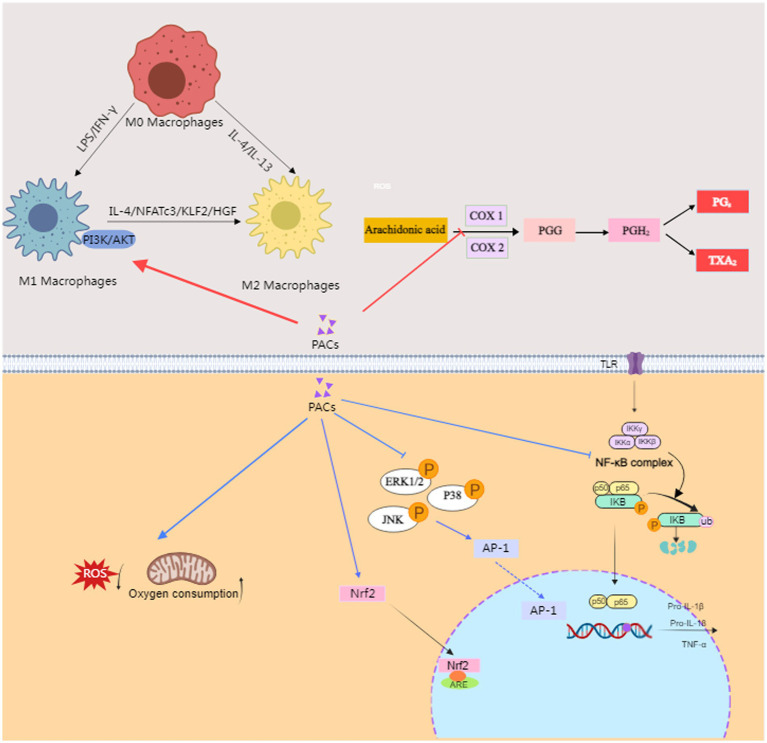
The anti-inflammatory mechanism of PACs. PACs, Proanthocyanidins; IFN, Interferon; IL, interleukin; NFATc3, Nuclear factors of activated T cells; KLF2, Krüppel-like factor 2; HGF, hepatocyte growth factor; PI3K, Phosphoinositide 3-kinase; AKT, Protein kinase B; COX, cyclooxygenase; PGG:G prostaglandin; PGH2, prostaglandin H2; PG2, prostacyclin PGI2; TXA2, A2 thromboxane A2; Nrf2, nuclear factor erythroid 2 related factor 2. ARE, antioxidant response element; ERK, extracellular regulated protein kinases; JNK, c-Jun N-terminal kinase; P38, Mitogen-Activated Protein Kinase 38; TLR, Toll-like receptor; NK-κB, nuclear factor-kappa B; TNF, tumor necrosis factor; This figure was conducted with medpeer (medpeer.cn).

When the body is subjected to damage by ROS and other oxidative stressors, these agents can trigger an inflammatory response, which is characterized by the infiltration of inflammatory cells and the release of inflammatory mediators. PACs mitigate this response by reducing the secretion of pro-inflammatory cytokines and enhancing the secretion of anti-inflammatory cytokines through the modulation of inflammatory pathways, thereby alleviating inflammation within the body. Moreover, PACs exert their therapeutic effects on ulcerative colitis in mice via the phosphatidylinositol 3-kinase (PI3K)/AKT/mammalian target of rapamycin (mTOR) pathway, which is a key signaling mechanism involved in the regulation of inflammation and immune responses (58). PACs modulate the inflammatory response by reducing the expression level of nuclear factor-κBp65 (NF-κBp65), which in turn lowers the level of inflammatory factors in the body (59, 60). They also regulate cell ferroptosis by activating the Nrf2/oxygenase-1(HO-1)/Kelch-like ECH-associated protein 1(Keap-1) signaling pathway, which is a critical endogenous antioxidant regulator (61). This activation leads to the translocation of Nrf2 to the nucleus, binding to antioxidant response element (ARE), and stimulating the transcription of antioxidant enzymes, thereby enhancing the body’s antioxidant capacity. Proanthocyanidin A1 has been shown to alleviate DSS-induced ulcerative colitis by regulating autophagy through the AMPK/mTOR/p70S6K signaling pathway (62). Proanthocyanidin B2 exerts its anti-inflammatory effects through a series of signaling pathways that involve the activation of protein kinase C (PKC) via PI3K, leading to the release of prostaglandin E2 (PGE2). This PGE2 then acts on the ATPase enzyme through the EP4 receptor and protein kinase A (PKA), thereby modulating the arachidonic acid pathway, which is central to the production of inflammatory mediators such as prostaglandins and leukotrienes (63). The activation of this pathway by Proanthocyanidin B2 suggests that it plays a role in regulating the inflammatory response and potentially alleviates inflammatory conditions like ulcerative colitis by influencing key enzymes and receptors involved in the arachidonic acid cascade. In addition, grape seed proanthocyanidins (GSP) have been shown to regulate M2a macrophage polarization through the TREM2/PI3K/Akt pathway, which plays a significant role in improving acute lung injury induced by lipopolysaccharide (64). This pathway is crucial for modulating the polarization of macrophages from a pro-inflammatory M1 phenotype to an anti-inflammatory M2a phenotype, which is beneficial in the context of acute lung injury. Grape seed proanthocyanidins (GSPE) have been shown to significantly reduce the expression of cyclooxygenase (COX)-2 by inactivating key signaling pathways involved in inflammation, specifically NF-κB and the ERK/P38 MAPK pathway (65). According to the research by Endo et al. (66), Proanthocyanidin B2 suppresses the production of interleukin (IL)-17 from T cells by selectively inhibiting cytokine production from dendritic cells (DCs). This suppression is mediated by first regulating the production of tumor necrosis factor (TNF)-*α* by CD4+ T cells and then inhibiting the secretion of IL-1β from DCs, which consequently leads to a decrease in IL-17 production. PACs from litchi have been found to enhance the infiltration of CD8+ cytotoxic T lymphocytes and decrease the quantity of macrophages, thereby modulating the tumor microenvironment and potentially augmenting the effectiveness of immunotherapies against colon cancer (67). In conclusion, the antioxidant properties of PACs likely constitute the foundational and intrinsic mechanisms of their anti-inflammatory effects. Beyond suppressing oxidative stress responses, PACs modulate inflammatory reactions through multiple pathways, including the regulation of the arachidonic acid pathway, the modulation of inflammation-related pathways and the polarization of macrophages (Figure 2).

### Protective effect on the intestines

Extensive research indicates that the consumption of PACs from various sources exerts a protective effect on intestinal tissue. These compounds confer potential benefits on the intestinal immune system through prebiotic effects and direct modulation of immune cell functions (68–70), thereby reducing ROS levels, enhancing antioxidant enzyme activities, and mitigating oxidative stress in the intestinal tissue (71, 72).

In animal models, the supplementation of PACs has been shown to decrease the feed: gain ratio (F/G), increase the prevalence of beneficial bacterial communities such as Akkermansia, Alistipes, and Bacteroides, and improve intestinal health. This improvement is primarily attributed to the enhancement of intestinal epithelial barrier function, increased apparent digestibility of nutrients, and upregulation of nutrient transport gene expression (73–75). Concurrently, PACs absorbed in the intestines undergo degradation and fermentation by intestinal microorganisms. Their metabolites, including phenyl *γ*-pentyl lactone and valeric acid, are further metabolized by the gut flora into phenolic acids. Upon intestinal absorption, these compounds are converted into glucuronic acid esters, methyl, or sulfate derivatives in the liver and are then excreted into the blood or ileum via bile.

### Lipid-regulating effects

Dyslipidemia is a principal determinant in the development of cardiovascular disease. PACs have demonstrated beneficial effects on lipid metabolism, including the regulation of circulating triglycerides (TG), total cholesterol (TC), high-density lipoprotein cholesterol (HDL-C), low-density lipoprotein cholesterol (LDL-C), and apolipoprotein A1 (Apo-A1) (76). As a result, PACs present a promising therapeutic strategy for the management of lipid metabolism disorders and potentially obesity. PACs have been shown to improve postprandial hypertriacylglycerolemia. It was indicated that GSPE elicits hypotriacylglycerolaemic effects by suppressing the secretion of lipoproteins (77). Another study demonstrated that PACs regulate lipid metabolism disorders by normalizing the intestinal microbiota structure, enhancing the intestinal barrier, inhibiting insulin resistance and oxidative stress, and alleviating liver inflammation and lipid accumulation (78). Lotus seedpod OPC affects the physicochemical properties of oil-in-water emulsions by reducing the particle size and viscosity of the emulsions. It inhibits lipase to suppress fat hydrolysis and lowers blood lipids (79).

### Glucose-regulating effect

Diabetes is a chronic metabolic disease where glucose metabolism is impaired. The glucose metabolism-modulating effect makes PACs important ingredients to produce functional foods for diabetics. An early study showed that dietary supplementation with cacao liquor PACs can dose-dependently prevent the development of hyperglycemia in diabetic obese mice (80). *In vivo* studies corroborate the hypothesis that the administration of proanthocyanidins (PACs) may ameliorate postprandial hyperglycemia in both rodents and humans. This effect is potentially mediated through the inhibition of pancreatic *α*-amylase (Amy) and α-glucosidase (Glu) (81, 82). PACs can bind to α-glucosidase, reducing α-helix reduction and increasing *β*-sheets, thereby intervening in the progression of type 2 diabetes by altering the conformation of α-glucosidase (83). PACs can lower the expression of intestinal glucose transporter protein in diabetic mice, thereby regulating serum metabolites and improving hyperglycemia (84). Moreover, non-enzymatic glycation of proteins can produce advanced glycation end products (AGEs) and important intermediates (such as methylglyoxal, α-dicarbonyl compounds), which are associated with diabetic complications and have potential health risks. PACs can directly inhibit this glycation product, alleviate diabetic complications (85).

### The effects on inhibiting cancer cell proliferation

PACs can directly inhibit the proliferation of cancer cells and induce apoptosis of cancer cells in a selective manner thereby exerting anti-tumor effects (85, 86). PC1 induces DNA damage cell cycle arrest and enhances the expression of Chk1 protein kinase reduces the level of Bcl-2 and increases the expression of BAX Caspase 3 and 9 in cancer cells exhibiting anti-proliferative characteristics against breast cancer cells through apoptosis induction (87). Studies have shown that procyanidin C1 PCB1 can induce apoptosis in cells at the S phase and cell cycle arrest by increasing the expression of pro-apoptotic protein caspase-3 and BAX and reducing the expression of anti-apoptotic protein Bcl-2. *In vivo* experiments using a xenograft mouse model have proven the effective anti-tumor activity of PCB1 indicating that PCB1 significantly inhibits tumor growth (88). PC reduces the proliferation of nude mouse CRC cells (HT29 and HCT-116) improves the sensitivity of CRC cells to oxaliplatin (L-OHP) and inhibits tumor growth. Further research shows that PC also down-regulates surface molecules and stemness transcription factors in CRC CSCs while inhibiting the formation of tumor spheroids and cell aggregates. In addition PC damaged the expression of p-gsk3*β* β-catenin and DVL1-3 proteins. PACs can activate Wnt/β-catenin signaling rescue the down-regulation of CSCs markers and reduce the formation ability of tumor spheroids and cell aggregates in CRC cells. PC exerts inhibitory effects on CSCs in colorectal cancer through Wnt/β-catenin which may be a potential natural drug for the treatment of colorectal cancer (89). In addition in the colon cancer model PACs significantly inhibit the metastasis of CT26 colon cancer cells by increasing the infiltration of CD8+ cytotoxic T lymphocytes and reducing the number of macrophages (67)

### Other biological effects

PACs inhibit xanthine oxidase (90) which can improve gout. PACs can inhibit chondrocyte apoptosis and improve arthritis (91). PACs improve cognitive impairment in mice showing potential application in Alzheimer’s disease (92). Additionally PACs have strong neuroprotective effects mainly through activation of the TLR4-p38-NF-κB-NLRP3 signaling pathway (93) or by activating the Nrf2/ARE pathway and alleviating oxidative damage (85). Proanthocyanidins inhibit renal mesangial cell dysfunction induced by hyperglycemia by regulating the cav-1-dependent signaling pathway (94).

## The application of PACs in pig production

### The effects of dietary PACs on growth performance in pigs

Growth performance directly reflects the growth condition of the pig herd and is the most valued intuitive indicator for the farm owners. With the implementation of antibiotic-free feeds, farm owners are showing more and more concerns on the growth of pigs in post-antibiotics age. In recent years, PACs have shown potential in antibiotics substitution, which supports the normal growth of pigs especially in poor conditions due to stress reaction or pathological condition. In pigs, studies have explored the effects of PACs on growth performance (Table 1). Most animal studies reflect that the effect of PACs on growth performance of pigs is limited (9, 95–97). During the weaning stage of piglets, supplementing the diet with an appropriate concentration of PACs can alleviate weaning stress in piglets, reduce the incidence of diarrhea, and improve production performance. By comparing the effects of grape seed procyanidins (GSP) with antibiotics on the growth performance, immune function and antioxidant capacity in weaned piglets, the study of Hao et al. (95) showed that the use of antibiotics or varying levels of GSP had no significant effects on average daily gain (ADG), average daily feed intake (ADFI) and F/G, whereas significantly reduced the incidence of diarrhea in piglets weaned at 21 days of age. Fang et al. (98) added 0, 40, 70, and 100 mg/kg of GSP to the weaning diets of piglets at 28 days of age, and the group with 40 mg/kg GSP showed significantly increased average daily gain and significantly reduced feed/gain ratio. In addition, the incidence of diarrhea in the 40 mg/kg GSP and 70 mg/kg GSP groups was significantly reduced, whereas all the groups supplemented with GSP had significantly increased activity of the digestive enzymes and antioxidant enzymes, which probably enhanced the body’s ability to resist weaning stress. Another study in weaned pigs indicated that the addition of PACs at 30 and 300 mg/kg to the diet significantly reduced the F:G and the incidence of diarrhea (99). Furthermore, PACs supplementation enhanced the apparent digestibility of dry matter, ether extract, gross energy, and ash. In another study supplementing 0.01 and 0.02% PACs in barrows’ diet, ADG and ADFI were not altered, whereas the G:F ratio increased significantly (100).

**Table 1:** The effects of dietary proanthocyanidins on growth performance and meat quality of pigs.

Source/form and dosage of PACs	Animals	Experiment duration (d)	Effect	References
Grape seed; 50, 100, 150 mg/kg	DLY piglets, 6.99 kg	28	ADG, ADFI and F/G were not affected	(95)
Grape seed; 0.01, 0.02%	Barrows, 19.2 kg	56	Increased G/F	(100)
Grape seed; 100, 200 mg/kg	castrated male DLY pigs, 94.8 kg	30	Increased loin eye area and improved meat color and tenderness. Increased slow myosin heavy chain (MyHC) expression in the LT muscle.	(101)
Grape seed; 50, 100, and 200 mg/kg.	Castrated DLY pigs, 67.47 kg	49	improved color, water-holding capacity, tenderness and nutritional value of pork, and increased percentage of slow-twitch fibers.	(102)
Grape seed; 150, 200, and 250 mg/kg.	DLY pigs, 30.85 kg	90	reduced low-density lipoprotein cholesterol and saturated fatty acid content, and increased unsaturated fatty acid content in LT mmuscle.	(103)
Grape seed; 50, 100, and 200 mg/kg.	Castrated pigs, 68.28 kg	49	ADG, ADFI and F/G were not affected	(96)
Grape seed; 250 mg/kg	Male and female Pigs (half half), 10.3 kg	28	ADG, ADFI and feed conversion were not affected	(9)
Grape seed; 250 mg/kg	Crossbred weaned piglets, 9.3 kg	28	Increased ADG, decreased F/G	(20)
Fruit by-product	Crossbred pigs, 27.94 kg	96	ADG, ADFI and F/G were not affected	(97)
Grape seed extract; 100, 300, and 700 mg/kg.	landrace × large white pigs, 46 kg	56	In raw LT steaks, surface lightness, redness, lipid stability and pH were not affected.	(105)

IBW, initial body weight; DLY, Duroc × Landrace × Yorkshine; LT, Longissimus thoracis.

### The effects of dietary PACs on meat quality in pigs

The positive impact of PACs on the meat quality of pigs has been substantiated through extensive research (Table 1). In a recent study conducted by Li et al. (101), GSP was supplemented to the diets of finishing pigs during the late stage of fattening and shown positive effects on pork quality. In this study, following the 30-day feeding trial, GSP significantly reduced backfat thickness, but increased loin eye area and improved meat color and tenderness. The supplementation of GSP in the diet activated AMPK through the NRF1 /Ca MKK *β* axis and regulated the transition of muscle fiber types through the AMPK/SIRT1/PGC-1 *α* signaling pathway, leading to the conversion of fast-twitch fibers to slow-twitch fibers, with GSP significantly reducing backfat thickness and increasing loin eye area, thereby improving meat color and tenderness. Xu et al. (102) added 0, 150, 200, and 250 mg/kg GSP to the finishing diet of fattening pigs and found that GSP could effectively improve the color, water-holding capacity, tenderness, and nutritional value of pork, increase the proportion of slow-twitch fibers in fattening pigs and their antioxidant capacity, thereby improving the sensory quality and nutritional value of pork, and that 200 mg/kg was the optimal addition amount. Zhang et al. (103) added 0, 150, 200, and 250 mg/kg GSP to the finishing diet of fattening pigs and found that different concentrations of GSPs in the diet upregulated the expression of CAB39, lipolysis LPL, and CD36 and CPT-1B involved in fatty acid transmembrane transport, while significantly reducing low-density lipoprotein cholesterol and saturated fatty acid content, and increasing unsaturated fatty acid content; the 200 or 250 mg/kg GSPs group significantly reduced the total cholesterol and triglyceride content in the longest thoracic muscle. Feng et al. (104) added 0, 100, and 200 mg/kg GSP to the finishing diet of fattening pigs and found that it upregulated the mRNA expression of genes related to fat decomposition and fatty acid oxidation, downregulated the mRNA expression of genes related to fat generation, and activated AMPK signaling; the GSPE supplement improved the antioxidant capacity and lipid metabolism of fattening pigs. Therefore, adding PACs during the finishing stage of fattening pigs can significantly improve the sensory evaluation and nutritional value of pork, while also reducing production costs, and has strong application potential in practical production. Nevertheless, the grape seed extract, when supplemented to pig diets at the tested levels, did not significantly improve the oxidative stability or quality of raw or cooked pork, nor did it reduce iron-induced lipid oxidation in tissue homogenates (105).

### The effects of dietary PACs on antioxidant capacity and immune status in pigs

The antioxidant activity is the most prominent function of PACs, and it has been proven to be capable of improving the oxidative stress in pigs (Table 2). In finishing pigs, the antioxidant assessment revealed that the addition of 200 mg/kg of grape seed proanthocyanidin extract (GSPE) to the diet significantly increased the levels of glutathione, overall antioxidant capacity, and glutathione peroxidase in serum, muscle, and liver tissues. Concurrently, this dietary intervention was associated with a notable decrease in the levels of malondialdehyde, a biomarker for oxidative stress (104). In another study in finishing pigs, dietary supplementation of GSPE at the dosages of 50 mg/kg, 100 mg/kg and 200 mg/kg have similar effects in improving the total antioxidant capacity and total superoxide dismutase and lowering the malondialdehyde content in longissimus dorsi muscle (102). In a study using weaned piglets as experimental subject, following a 21-day feeding trial, the plasma malondialdehyde concentration was reduced in piglets fed diets supplemented with polyphenols mixture, which containing 27.5% grape seed polyphenol (106).

**Table 2:** The effects of dietary proanthocyanidins on antioxidant capacity and immune status in pigs.

Source/form and dosage of PACs	Animals and IBW	Experiment duration (d)	Effect	References
Grape seed; 50, 100, 150 mg/kg	DLY piglets, 6.99 kg	28	Higher T-AOC and GSH-Px and lower MDA in serum; Pigs consumed GSP generated higher serum IgG, IgM, C4 and IL-2 concentrations.	(95)
Grape seed; 40, 70, 100 mg/kg	Pietrain × large white piglets, 8.4 kg	96	increased the activities of GSH-Px, SOD and T-AOC in serum, liver and muscle	(98)
Grape seed; 50, 100, 150 mg/kg	DLY piglets, 6.52 kg	28	reduced the serum DAO activity and decreased the plasma endotoxin levels	(111)
Grape seed; 0.02, 0.04%	Barrows, 13.4 kg	28	Secretion of IL-1β, IL-6 and TNF-α by cultured peripheral blood mononuclear cells from pigs challenged with LPS were lower	(100)
Grape seed; 100, 200 mg/kg	castrated Duroc × Landrace × Yorkshire (DLY) pigs	30	Increased GSH, T-AOC and GSH-Px levels, and reduced MDA levels in serum, muscle and liver	(104)
Grape seed; 50, 100, 200 mg/kg.	Castrated pigs, 68.28 kg	49	Lower MDA in serum and liver, higher GSH-Px in serum and liver	(96)
Grape seed; 250 mg/kg	Male and female Pigs (half half), 10.3 kg	28	Lower MDA and higher SOD in adipose tissue lower MCP-1, IL-6 and TNF-α	(9)
Citrus peel/ Persimmon peel	27.94 kg	96	Increased Ig A and IgM secretion after LPS challenge; increased the SOD-like activity	(97)
Grape seed meal	TOPIGS-40 crossbred piglets, 9.0 kg	30	Reduced proinflammatory MMP-2 and MMP-9 genes and activities; suppressed expression of the innate immune TLR-2 and TLR-4 genes	(107)
Grape, 5%	helminth-naive pigs, 21 kg	24	increased numbers of eosinophils induced by Ascaris suum infection in the duodenum, jejunum and ileum, and modulated gene expression in the jejunal mucosa of infected pigs	(115)

IBW, initial body weight; DLY, Duroc × Landrace × Yorkshine; MDA, malondialdehyde; SOD, superoxide dismutase; GSH, glutathione; GSH-Px, glutathione peroxidase; MCP-1, monocyte chemoattractant protein-1; MMP, matrix metalloproteinase; TLR, Toll-like receptor; Ig, immunoglobulin; C4, complement 4; TNF, tumor necrosis factor; IL, interleukin.

Inflammatory response, especially intestinal inflammation, is quite a common pathological condition in pig production as a consequence of existing environmental pathogens. *In vivo* and *in vitro* studies have shown the anti-inflammatory effects of PACs in pigs. Weaned piglets that were given diets supplemented with GSP at levels of 100 or 150 mg/kg exhibited significantly higher serum concentrations of immunoglobulins (IgG and IgM), complement 4 (C4), and interleukin-2 (IL-2) (95). In pigs consumed diets containing 0.04% PACs, significantly lower platelet count was observed 24 h after an LPS challenge (100). In pigs with DSS induced colitis, the diet supplemented with grape seed meal effectively counteracted inflammatory disorders by inhibiting the activation of MAPKs, attenuating the expression of NF-κB at both the gene and protein levels, and reducing the production of pro-inflammatory cytokines and chemokines to control levels in DSS-treated piglets (107). *In vitro* study using Peripheral blood mononuclear cells (PBMCs) from pigs demonstrated that the secretion of cytokines including IL-1β, IL-6, and TNF-*α* in the presence of procyanidin was significantly lower compared to PBS at 4 h post-LPS challenge (*p* < 0.05). Fiesel et al. (108) showed that pigs fed grape seed and grape marc meal extract had a lower expression of various pro-inflammatory genes in duodenum, ileum and colon than the control group. The intestinal barrier integrity is of great importance in decreasing the incidence of inflammatory responses (109, 110). In weaned piglets, supplementation of 250 mg/kg PACs had the potential to enhance intestinal barrier function as indicated by the decreased intestinal permeability (20). These studies indicated the potential anti-inflammatory effect of PACs (Table 2).

### The effects of dietary PACs on gut physiology and microbiota in pigs

Li et al. (111) found that higher concentrations of GSPs in the diet reduced the digestion of nutrients in piglets, inhibited the activity of digestive enzymes; appropriate concentrations of GSPs can promote the activity of intestinal brush border enzymes and reduce epithelial permeability. Notably, proanthocyanidins (PACs) not only significantly increased the villus height and the ratio of villus height to crypt depth (V/C) in both the duodenum and jejunum, but also caused a significant reduction in crypt depth within the duodenum (99). Furthermore, the administration of PACs at dosages of 30, 300, and 600 mg/kg enhanced the expression levels of mucin 1, mucin 2, and fatty acid transport protein 1 in the duodenum. Likewise, PACs supplementation upregulated the expression of fatty acid transport protein 4 in both the jejunum and ileum (99). It was proposed by Wei et al. that GSPs could positively influence the intestinal health of weaned piglets. This was achieved by enhancing intestinal antioxidant capacity, modulating the intestinal microbiota composition, and elevating the levels of intestinal microbial metabolites, specifically short-chain fatty acids (112).

The interplay between the intestinal microbiota and gastrointestinal health has become a focal point of research, with implications for enhancing the productivity and welfare of swine in the context of livestock farming (113). PACs are suggested to exert beneficial effects on metabolic homeostasis by modulating the gut microbiota and intestinal metabolic functions (114). Compared with Bama minipigs fed antibiotics, these pigs fed PACs exhibited less abundant genera Fibrobacter and Spirochaete. Principal component analysis revealed clear separations between antibiotic and PACs groups (114). PACs consumption has been linked to better colonic health, but PACs are poorly absorbed, making them a target for colonic metabolism (21). The dietary supplementation with grape seed extract (GSE) in sows was reported to induce a significant ecological perturbation within the intestinal microbiota, characterized by a pronounced augmentation in the relative abundance of key bacterial taxa, including the Lachnospiraceae family, the Clostridiales order, the Lactobacillus genus and the Ruminococcaceae family (21). A diet enriched with PACs has been reported to significantly alter the composition of the prokaryotic gut microbiota and reduce the concentrations of isobutyric and isovaleric acids in the colon, thereby modulating the host’s subsequent response to helminth infections (115). These studies demonstrated the significant effects of PACs in regulating gut microbiota and host immunity.

## Conclusion

PACs are widely existing polyphenolic substances in nature. Currently the extraction and purification technologies are mature and the obtained products have high purity and low cost. PACs have various biological activities such as antioxidant anti-inflammatory anti-cancer hypoglycemic and fat metabolism improvement which endows them with broad development potential in pig production under the background of a complete ban on antibiotics. Adding PACs to the diet during the weaning period of suckling pigs can repair the intestinal barrier reduce the diarrhea index of suckling pigs increase the average daily weight gain and reduce the feed-to-weight ratio. In the fattening stage of pigs whether it is the early stage or the later stage of short-term feeding with PACs it can improve the meat quality and increase its nutritional value. However there is still a lack of research on the reproductive performance of sows with PACs and further exploration is needed. In conclusion the use of PACs as a growth promoter in pig production is a major stride toward finding alternatives to antibiotics.

## References

[1] Low CX Tan LT-H Ab Mutalib NS Pusparajah P Goh BH Chan KG. Unveiling the impact of antibiotics and alternative methods for animal husbandry: a review. Antibiotics. (2021) 10:578. doi: 10.3390/antibiotics10050578, PMID: 34068272 PMC8153128

[2] Liu S Wang K Lin S Zhang Z Cheng M Hu S. Comparison of the effects between tannins extracted from different natural plants on growth performance, antioxidant capacity, immunity, and intestinal Flora of broiler chickens. Antioxidants. (2023) 12:441. doi: 10.3390/antiox12020441, PMID: 36829999 PMC9952188

[3] Shirzadi H Shariatmadari F Karimi Torshizi MA Rahimi S Masoudi AA Zaboli G. Plant extract supplementation as a strategy for substituting dietary antibiotics in broiler chickens exposed to low ambient temperature. Arch Anim Nutr. (2020) 74:206–21. doi: 10.1080/1745039X.2019.1693860, PMID: 31852306

[4] Alem WT. Effect of herbal extracts in animal nutrition as feed additives. Heliyon. (2024) 10:e24973. doi: 10.1016/j.heliyon.2024.e24973, PMID: 38322944 PMC10845724

[5] Dabbou S Gasco L Rotolo L Pozzo L Tong JM Dong XF. Effects of dietary alfalfa flavonoids on the performance, meat quality and lipid oxidation of growing rabbits. Asian Australas J Anim Sci. (2018) 31:270–7. doi: 10.5713/ajas.17.0284, PMID: 28728357 PMC5767510

[6] Liu Y Xiao Y Xie J Peng Y Li F Chen C. Dietary supplementation with flavonoids from mulberry leaves improves growth performance and meat quality, and alters lipid metabolism of skeletal muscle in a Chinese hybrid pig. Anim Feed Sci Technol. (2022) 285:115211. doi: 10.1016/j.anifeedsci.2022.115211

[7] Biswas S Kim IH. Assessment of Quillaja saponin as a feed supplement in maize-soybean-oilseed rape meal-based diet for enhanced growing pig performance. J Anim Feed Sci. (2024) 33:193–9. doi: 10.22358/jafs/171702/2023

[8] Liu X Le Bourvellec C Guyot S Renard CM. Reactivity of flavanols: their fate in physical food processing and recent advances in their analysis by depolymerization. Compr Rev Food Sci Food Saf. (2021) 20:4841–80. doi: 10.1111/1541-4337.12797, PMID: 34288366

[9] Wu Y Ma N Song P He T Levesque C Bai Y. Grape seed Proanthocyanidin affects lipid metabolism via changing gut microflora and enhancing propionate production in weaned pigs. J Nutr. (2019) 149:1523–32. doi: 10.1093/jn/nxz102, PMID: 31175811

[10] Xu Y Huang Y Chen Y Cao K Liu Z Wan Z. Grape seed Proanthocyanidins play the roles of radioprotection on Normal lung and radiosensitization on lung Cancer via differential regulation of the MAPK signaling pathway. J Cancer. (2021) 12:2844–54. doi: 10.7150/jca.49987, PMID: 33854585 PMC8040900

[11] Chen L Hu SL Xie J Yan DY Weng SJ Tang JH. Proanthocyanidins-mediated Nrf2 activation ameliorates glucocorticoid-induced oxidative stress and mitochondrial dysfunction in osteoblasts. Oxidative Med Cell Longev. (2020) 2020:9102012–4. doi: 10.1155/2020/9102012, PMID: 33062149 PMC7533007

[12] Zhang M Mo R Wang H Liu T Zhang G Wu Y. Grape seed proanthocyanidin improves intestinal inflammation in canine through regulating gut microbiota and bile acid compositions. FASEB J. (2023) 37:e23285. doi: 10.1096/fj.202300819RR 37933950

[13] Niedzwiecki A Roomi MW Kalinovsky T Rath M. Anticancer efficacy of polyphenols and their combinations. Nutrients. (2016) 8:552. doi: 10.3390/nu8090552, PMID: 27618095 PMC5037537

[14] Han X Shen T Lou H. Dietary polyphenols and their biological significance. Int J Mol Sci. (2007) 8:950–88. doi: 10.3390/i8090950

[15] Zeng Y Zhao L Wang K Renard CMGC le Bourvellec C Hu Z. A-type proanthocyanidins: sources, structure, bioactivity, processing, nutrition, and potential applications. Compr Rev Food Sci Food Saf. (2024) 23:e13352. doi: 10.1111/1541-4337.13352, PMID: 38634188

[16] Liu C Bolling BW. Dietary proanthocyanidins for improving gut immune health. Curr Opin Food Sci. (2024) 56:101133. doi: 10.1016/j.cofs.2024.101133

[17] Zhu W Oteiza PI. Proanthocyanidins at the gastrointestinal tract: mechanisms involved in their capacity to mitigate obesity-associated metabolic disorders. Crit Rev Food Sci Nutr. (2024) 64:220–40. doi: 10.1080/10408398.2022.2105802, PMID: 35943169

[18] de Araújo FM Alves Carnauba R Fernandes GA Pimentel de Assumpção P Curado MP. Polyphenol intake and gastric cancer: a case-control study in the Brazilian Amazon region. Cancer Epidemiol. (2024) 88:102518. doi: 10.1016/j.canep.2023.102518, PMID: 38171205

[19] Bondonno NP Parmenter BH Murray K Bondonno CP Blekkenhorst LC Wood AC. Associations between flavonoid intake and subclinical atherosclerosis: the multi-ethnic study of atherosclerosis. Arterioscler Thromb Vasc Biol. (2024) 44:2347–59. doi: 10.1161/ATVBAHA.124.321106, PMID: 39263763

[20] Han M Song P Huang C Rezaei A Farrar S Brown MA. Dietary grape seed proanthocyanidins (GSPs) improve weaned intestinal microbiota and mucosal barrier using a piglet model. Oncotarget. (2016) 7:80313–26. doi: 10.18632/oncotarget.13450, PMID: 27880936 PMC5348322

[21] Choy YY Quifer-Rada P Holstege DM Frese SA Calvert CC Mills DA. Phenolic metabolites and substantial microbiome changes in pig feces by ingesting grape seed proanthocyanidins. Food Funct. (2014) 5:2298–308. doi: 10.1039/c4fo00325j, PMID: 25066634 PMC4744461

[22] Baila C Lobón S Blanco M Casasús I Ripoll G Joy M. Sainfoin in the dams’ diet as a source of Proanthocyanidins: effect on the growth, carcass and meat quality of their suckling lambs. Animals. (2022) 12:408. doi: 10.3390/ani12040408, PMID: 35203116 PMC8868129

[23] Wang ML Suo X Gu JH Zhang WW Fang Q Wang X. Influence of grape seed Proanthocyanidin extract in broiler chickens: effect on chicken coccidiosis and antioxidant status. Poult Sci. (2008) 87:2273–80. doi: 10.3382/ps.2008-00077, PMID: 18931178

[24] Park I-J Cha S-Y Kang M Jang H-K. Immunomodulatory effect of a proanthocyanidin-rich extract from *Pinus radiata* bark by dosing period in chickens. Poult Sci. (2013) 92:352–7. doi: 10.3382/ps.2012-02704, PMID: 23300300

[25] Roux EL Doco T Sarni-Manchado P Lozano Y Cheynier V. A-type proanthocyanidins from pericarp of *Litchi chinensis*. Phytochemistry. (1998) 48:1251–8. doi: 10.1016/S0031-9422(97)01070-4

[26] Pasini F Chinnici F Caboni MF Verardo V. Recovery of oligomeric Proanthocyanidins and other phenolic compounds with established bioactivity from grape seed by-products. Molecules. (2019) 24:677. doi: 10.3390/molecules24040677, PMID: 30769803 PMC6413075

[27] Ma Z Huang Y Huang W Feng X Yang F Li D. Separation, identification, and antioxidant activity of polyphenols from Lotus seed Epicarp. Molecules. (2019) 24:4007. doi: 10.3390/molecules24214007, PMID: 31694314 PMC6864829

[28] Irakli M Lazaridou A Mylonas I Biliaderis CG. Bioactive components and antioxidant activity distribution in pearling fractions of different Greek barley cultivars. Food Secur. (2020) 9:783. doi: 10.3390/foods9060783, PMID: 32545662 PMC7353517

[29] Qin Z Liu H-M Ma Y-X Wang X-D. Chapter 9- developments in extraction, purification, and structural elucidation of proanthocyanidins (2000–2019) In: Atta Ur R, editor. Studies in natural products chemistry. Amsterdam: Elsevier (2021). 347–91.

[30] Gu L Kelm MA Hammerstone JF Beecher G Holden J Haytowitz D. Screening of foods containing proanthocyanidins and their structural characterization using LC-MS/MS and thiolytic degradation. J Agric Food Chem. (2003) 51:7513–21. doi: 10.1021/jf034815d, PMID: 14640607

[31] Sharma PK Romanczyk LJ Jr Kondaveti L Reddy B Arumugasamy J Lombardy R. Total synthesis of proanthocyanidin A1, A2, and their stereoisomers. Org Lett. (2015) 17:2306–9. doi: 10.1021/acs.orglett.5b00646, PMID: 25927567

[32] Ye H Luo L Wang J Jiang K Yue T Yang H. Highly galloylated and A-type prodelphinidins and procyanidins in persimmon (*Diospyros kaki* L.) peel. Food Chem. (2022) 378:131972. doi: 10.1016/j.foodchem.2021.131972, PMID: 35032795

[33] Zhao L Yan F Lu Q Tang C Wang X Liu R. UPLC-Q-TOF-MS and NMR identification of structurally different A-type procyanidins from peanut skin and their inhibitory effect on acrylamide. J Sci Food Agric. (2022) 102:7062–71. doi: 10.1002/jsfa.12067, PMID: 35690888

[34] Chen J Chen Y Zheng Y Zhao J Yu H Zhu J. Relationship between neuroprotective effects and structure of Procyanidins. Molecules. (2022) 27:27. doi: 10.3390/molecules27072308, PMID: 35408708 PMC9000754

[35] Acosta-Otalvaro E Valencia-Gallego W Mazo-Rivas JC Garcia-Viguera C. Cocoa extract with high content of flavan 3-ols, procyanidins and methylxanthines. J Food Sci Technol. (2022) 59:1152–61. doi: 10.1007/s13197-021-05119-z, PMID: 35153329 PMC8814059

[36] Miranda-Hernández AM Muñiz-Márquez DB Wong-Paz JE Aguilar-Zárate P de la Rosa-Hernández M Larios-Cruz R. Characterization by HPLC-ESI-MS (2) of native and oxidized procyanidins from litchi (*Litchi chinensis*) pericarp. Food Chem. (2019) 291:126–31. doi: 10.1016/j.foodchem.2019.04.020, PMID: 31006450

[37] Elshibani FA Alamami AD Mohammed HA Rasheed RA el Sabban RM Yehia MA. A multidisciplinary approach to the antioxidant and hepatoprotective activities of Arbutus pavarii Pampan fruit; in vitro and in vivo biological evaluations, and in silico investigations. J Enzyme Inhib Med Chem. (2024) 39:2293639. doi: 10.1080/14756366.2023.2293639, PMID: 38153110 PMC10763860

[38] Enomoto H Takahashi S Takeda S Hatta H. Distribution of Flavan-3-ol species in ripe strawberry fruit revealed by matrix-assisted laser desorption/ionization-mass spectrometry imaging. Molecules. (2019) 25:25. doi: 10.3390/molecules25010103, PMID: 31888096 PMC6982903

[39] Ma JN Feng X Shan CB Ma Y Lu ZY Zhang DJ. Quantification and purification of procyanidin B1 from food byproducts. J Food Sci. (2022) 87:4905–16. doi: 10.1111/1750-3841.16358, PMID: 36303405

[40] Longo E Rossetti F Jouin A Teissedre PL Jourdes M Boselli E. Distribution of crown hexameric procyanidin and its tetrameric and pentameric congeners in red and white wines. Food Chem. (2019) 299:125125. doi: 10.1016/j.foodchem.2019.125125, PMID: 31299515

[41] Merkytė V Longo E Jourdes M Jouin A Teissedre PL Boselli E. High-performance liquid chromatography-hydrogen/deuterium exchange-high-resolution mass spectrometry partial identification of a series of tetra- and Pentameric cyclic Procyanidins and Prodelphinidins in wine extracts. J Agric Food Chem. (2020) 68:3312–21. doi: 10.1021/acs.jafc.9b06195, PMID: 31930914 PMC7993638

[42] Laitila JE Salminen JP. Quantitative and qualitative composition of proanthocyanidins and other polyphenols in commercial red wines and their contribution to sensorially evaluated tannicity. Food Res Int. (2024) 177:113867. doi: 10.1016/j.foodres.2023.113867, PMID: 38225134

[43] Lv T-T Qin Z Liu H-M Wang X-D He J-R. Chemical composition and antioxidant capacity of proanthocyanidins from Chinese quince (*Chaenomeles sinensis*) fruit at different growth stages. J Food Meas Charact. (2024) 18:2318–30. doi: 10.1007/s11694-023-02314-8

[44] Zhu QY Holt RR Lazarus SA Ensunsa JL Hammerstone JF Schmitz HH. Stability of the Flavan-3-ols Epicatechin and Catechin and related dimeric Procyanidins derived from cocoa. J Agric Food Chem. (2002) 50:1700–5. doi: 10.1021/jf011228o, PMID: 11879061

[45] Khanal RC Howard LR Prior RL. Procyanidin composition of selected fruits and fruit byproducts is affected by extraction method and variety. J Agric Food Chem. (2009) 57:8839–43. doi: 10.1021/jf9015398, PMID: 19722520

[46] Sun L Wang H Du J Wang T Yu D. Ultrasonic-assisted extraction of grape seed procyanidins, preparation of liposomes, and evaluation of their antioxidant capacity. Ultrason Sonochem. (2024) 105:106856. doi: 10.1016/j.ultsonch.2024.106856 38554530 PMC10995857

[47] Chen J Thilakarathna WPDW Astatkie T Rupasinghe HPV. Optimization of Catechin and Proanthocyanidin recovery from grape seeds using microwave-assisted extraction. Biomol Ther. (2020) 10:243. doi: 10.3390/biom10020243 PMC7072399 32033405

[48] Fernández K Vega M Aspé E. An enzymatic extraction of proanthocyanidins from País grape seeds and skins. Food Chem. (2015) 168:7–13. doi: 10.1016/j.foodchem.2014.07.021, PMID: 25172676

[49] Gu L Kelm M Hammerstone JF Beecher G Cunningham D Vannozzi S. Fractionation of polymeric Procyanidins from lowbush blueberry and quantification of Procyanidins in selected foods with an optimized Normal-phase HPLC−MS fluorescent detection method. J Agric Food Chem. (2002) 50:4852–60. doi: 10.1021/jf020214v, PMID: 12166971

[50] Sui Y Zheng Y Li X Li S Xie B Sun Z. Characterization and preparation of oligomeric procyanidins from *Litchi chinensis* pericarp. Fitoterapia. (2016) 112:168–74. doi: 10.1016/j.fitote.2016.06.001, PMID: 27282208

[51] Zhang S Li L Cui Y Luo L Li Y Zhou P. Preparative high-speed counter-current chromatography separation of grape seed proanthocyanidins according to degree of polymerization. Food Chem. (2017) 219:399–407. doi: 10.1016/j.foodchem.2016.09.170, PMID: 27765243

[52] Han X Zhou Q Gao Z Bianca Xu G Chen H Chitrakar B. Characterization of procyanidin extracts from hawthorn (*Crataegus pinnatifida*) in human colorectal adenocarcinoma cell line Caco-2, simulated digestion, and fermentation identified unique and novel prebiotic properties. Food Res Int. (2023) 165:112393. doi: 10.1016/j.foodres.2022.112393 36869464

[53] Justino AB Franco RR Silva HCG Saraiva AL Sousa RMF Espindola FS. B procyanidins of Annona crassiflora fruit peel inhibited glycation, lipid peroxidation and protein-bound carbonyls, with protective effects on glycated catalase. Sci Rep. (2019) 9:19183. doi: 10.1038/s41598-019-55779-3, PMID: 31844118 PMC6915705

[54] Bayer J Hogger P. Development and validation of a LC-MS/MS method for the quantification of phenolic compounds in human saliva after intake of a procyanidin-rich pine bark extract. J Pharm Biomed Anal. (2024) 239:115914. doi: 10.1016/j.jpba.2023.115914, PMID: 38101241

[55] Zhang L Guan Q Zhang H Tang L Xu M. A spectroscopic and quantum chemical calculation method for the characterisation of metal ions complexed with propyl gallate and procyanidins. Sci Rep. (2023) 13:2977. doi: 10.1038/s41598-023-30186-x, PMID: 36806744 PMC9941574

[56] Wang H Hao W Yang L Li T Zhao C Yan P. Procyanidin B2 alleviates heat-induced oxidative stress through the Nrf2 pathway in bovine mammary epithelial cells. Int J Mol Sci. (2022) 23:23. doi: 10.3390/ijms23147769, PMID: 35887117 PMC9316217

[57] Chen L Hao L Yanshuo C FangFang W Daqin C Weidong X. Grape seed proanthocyanidins regulate mitophagy of endothelial cells and promote wound healing in mice through p-JNK/FOXO3a/ROS signal pathway. Arch Biochem Biophys. (2023) 749:109790. doi: 10.1016/j.abb.2023.109790, PMID: 37858664

[58] Huang B Wang L Liu M Wu X Lu Q Liu R. The underlying mechanism of A-type procyanidins from peanut skin on DSS-induced ulcerative colitis mice by regulating gut microbiota and metabolism. J Food Biochem. (2022) 46:e14103. doi: 10.1111/jfbc.14103, PMID: 35218055

[59] Lobo A Liu Y Song Y Liu S Zhang R Liang H. Effect of procyanidins on lipid metabolism and inflammation in rats exposed to alcohol and iron. Heliyon. (2020) 6:e04847. doi: 10.1016/j.heliyon.2020.e04847, PMID: 32964156 PMC7490533

[60] Hu L An E Zhu Z Cai Y Ye X Zhou H. Grape seed-derived procyanidins decreases neuropathic pain and nerve regeneration by suppression of toll-like receptor 4-myeloid differentiation factor-88 signaling. Mol Pain. (2024) 20:17448069241256466. doi: 10.1177/17448069241256466, PMID: 38716504 PMC11110500

[61] Zeng J Weng Y Lai T Chen L Li Y Huang Q. Procyanidin alleviates ferroptosis and inflammation of LPS-induced RAW264.7 cell via the Nrf2/HO-1 pathway. Naunyn Schmiedeberg's Arch Pharmacol. (2024) 397:4055–67. doi: 10.1007/s00210-023-02854-2, PMID: 38010399

[62] Zhang H Lang W Liu X Bai J Jia Q Shi Q. Procyanidin A1 alleviates DSS-induced ulcerative colitis via regulating AMPK/mTOR/p70S6K-mediated autophagy. J Physiol Biochem. (2022) 78:213–27. doi: 10.1007/s13105-021-00854-5, PMID: 35001346

[63] Zeineddine S Kreydiyyeh S. The signaling pathway through which Procyanidin B2 increases the activity of the Na+/K+ ATPase in Caco-2 cells. J Funct Foods. (2024) 119:106301. doi: 10.1016/j.jff.2024.106301

[64] Qiao X Wang H He Y Song D Altawil A Wang Q. Grape seed Proanthocyanidin ameliorates LPS-induced acute lung injury by modulating M2a macrophage polarization via the TREM2/PI3K/Akt pathway. Inflammation. (2023) 46:2147–64. doi: 10.1007/s10753-023-01868-5, PMID: 37566293 PMC10673742

[65] Chen WC Hossen M Liu W Yen CH Huang CH Hsu YC. Grape seed Proanthocyanidins inhibit replication of the dengue virus by targeting NF-kB and MAPK-mediated Cyclooxygenase-2 expression. Viruses. (2023) 15:15. doi: 10.3390/v15040884, PMID: 37112864 PMC10140912

[66] Endo K Matsui R Asami T Sawa T Nakashima A Tanaka Y. The suppression of IL-17 production from T cells by gallate-type procyanidin is mediated by selectively inhibiting cytokine production from dendritic cells. Biomed Pharmacother. (2021) 137:111346. doi: 10.1016/j.biopha.2021.111346, PMID: 33556876

[67] Yao Y Feng S Li X Liu T Ye S Ma L. Litchi procyanidins inhibit colon cancer proliferation and metastasis by triggering gut-lung axis immunotherapy. Cell Death Dis. (2023) 14:109. doi: 10.1038/s41419-022-05482-5, PMID: 36774343 PMC9922286

[68] Andersen-Civil AIS Arora P Williams AR. Regulation of enteric infection and immunity by dietary Proanthocyanidins. Front Immunol. (2021) 12:637603. doi: 10.3389/fimmu.2021.637603, PMID: 33717185 PMC7943737

[69] Ganesan K Quiles JL Daglia M Xiao J Xu B. Dietary phytochemicals modulate intestinal epithelial barrier dysfunction and autoimmune diseases. Food Front. (2021) 2:357–82. doi: 10.1002/fft2.102

[70] Endo K Sawa T Tanaka Y Saiki T Haga H Rizeq L. Oral administration of procyanidin B2 3,3″-di-O-gallate ameliorates experimental autoimmune encephalomyelitis through immunosuppressive effects on CD4(+) T cells by regulating glycolysis. Eur J Pharmacol. (2023) 954:175879. doi: 10.1016/j.ejphar.2023.175879, PMID: 37406847

[71] Gil-Cardoso K Ginés I Pinent M Ardévol A Arola L Blay M. Chronic supplementation with dietary proanthocyanidins protects from diet-induced intestinal alterations in obese rats. Mol Nutr Food Res. (2017) 61:61. doi: 10.1002/mnfr.201601039, PMID: 28218448

[72] Gil-Cardoso K Comitato R Ginés I Ardévol A Pinent M Virgili F. Protective effect of Proanthocyanidins in a rat model of mild intestinal inflammation and impaired intestinal permeability induced by LPS. Mol Nutr Food Res. (2019) 63:e1800720. doi: 10.1002/mnfr.201800720, PMID: 30656830

[73] Wang W Zhan L Guo D Xiang Y Tian M Zhang Y. Grape seed proanthocyanidins inhibit proliferation of pancreatic cancer cells by modulating microRNA expression. Oncol Lett. (2019) 17:2777–87. doi: 10.3892/ol.2019.9887, PMID: 30854052 PMC6365901

[74] Nallathambi R Poulev A Zuk JB Raskin I. Proanthocyanidin-rich grape seed extract reduces inflammation and oxidative stress and restores tight junction barrier function in Caco-2 Colon cells. Nutrients. (2020) 12:12. doi: 10.3390/nu12061623, PMID: 32492806 PMC7352846

[75] Wang N Chen W Cui C Zheng Y Yu Q Ren H. The Peanut skin Procyanidins attenuate DSS-induced ulcerative colitis in C57BL/6 mice. Antioxidants. (2022) 11:11. doi: 10.3390/antiox11112098, PMID: 36358470 PMC9686776

[76] Wang P Liu XL Jiang ZZ Long Y Gao CL Huang W. Effect of proanthocyanidins on blood lipids: a systematic review and meta-analysis. Phytother Res. (2024) 38:2154–64. doi: 10.1002/ptr.8162, PMID: 38391003

[77] Quesada H Díaz S Pajuelo D Fernández-Iglesias A Garcia-Vallvé S Pujadas G. The lipid-lowering effect of dietary proanthocyanidins in rats involves both chylomicron-rich and VLDL-rich fractions. Br J Nutr. (2012) 108:208–17. doi: 10.1017/S0007114511005472, PMID: 22011563

[78] Han X Zhao W Zhou Q Chen H Yuan J Xiaofu Z. Procyanidins from hawthorn (*Crataegus pinnatifida*) alleviate lipid metabolism disorder via inhibiting insulin resistance and oxidative stress, normalizing the gut microbiota structure and intestinal barrier, and further suppressing hepatic inflammation and lipid accumulation. Food Funct. (2022) 13:7901–17. doi: 10.1039/d2fo00836j, PMID: 35781311

[79] Li X Chen Y Li S Chen M Xiao J Xie B. Oligomer Procyanidins from Lotus seedpod regulate lipid homeostasis partially by modifying fat emulsification and digestion. J Agric Food Chem. (2019) 67:4524–34. doi: 10.1021/acs.jafc.9b01469, PMID: 30945544

[80] Tomaru M Takano H Osakabe N Yasuda A Inoue KI Yanagisawa R. Dietary supplementation with cacao liquor proanthocyanidins prevents elevation of blood glucose levels in diabetic obese mice. Nutrition. (2007) 23:351–5. doi: 10.1016/j.nut.2007.01.007, PMID: 17350804

[81] Zhou P Zhang L Li W Zhang S Luo L Wang J. In vitro evaluation of the anti-digestion and antioxidant effects of grape seed procyanidins according to their degrees of polymerization. J Funct Foods. (2018) 49:85–95. doi: 10.1016/j.jff.2018.08.001

[82] Tsujita T Shintani T Sato H. α-Amylase inhibitory activity from nut seed skin polyphenols. 1. Purification and characterization of almond seed skin polyphenols. J Agric Food Chem. (2013) 61:4570–6. doi: 10.1021/jf400691q, PMID: 23614772

[83] Zhao L Wen L Lu Q Liu R. Interaction mechanism between α-glucosidase and A-type trimer procyanidin revealed by integrated spectroscopic analysis techniques. Int J Biol Macromol. (2020) 143:173–80. doi: 10.1016/j.ijbiomac.2019.12.021, PMID: 31816382

[84] Liu M Shen J Zhu X Ju T Willing BP Wu X. Peanut skin procyanidins reduce intestinal glucose transport protein expression, regulate serum metabolites and ameliorate hyperglycemia in diabetic mice. Food Res Int. (2023) 173:113471. doi: 10.1016/j.foodres.2023.113471, PMID: 37803795

[85] Zeng YX Wang S Wei L Cui YY Chen YH. Proanthocyanidins: components, pharmacokinetics and biomedical properties. Am J Chin Med. (2020) 48:813–69. doi: 10.1142/s0192415x2050041x, PMID: 32536248

[86] Osorio-Cruz Y Olivares-Corichi IM Correa-Basurto J González-Garrido JA Pereyra-Vergara F Rivera G. The autoxidized mixture of (−)-Epicatechin contains Procyanidins and shows Antiproliferative and apoptotic activity in breast Cancer cells. Pharmaceuticals (Basel). (2024) 17:17. doi: 10.3390/ph17020258, PMID: 38399473 PMC10892779

[87] Koteswari LL Kumari S Kumar AB Malla RR. A comparative anticancer study on procyanidin C1 against receptor positive and receptor negative breast cancer. Nat Prod Res. (2020) 34:3267–74. doi: 10.1080/14786419.2018.1557173, PMID: 30618284

[88] Lei Y Deng X Zhang Z Chen J. Natural product procyanidin B1 as an antitumor drug for effective therapy of colon cancer. Exp Ther Med. (2023) 26:506. doi: 10.3892/etm.2023.12205, PMID: 37822589 PMC10562962

[89] Chen Y Yang Z He X Zhu W Wang Y Li J. Proanthocyanidins inhibited colorectal cancer stem cell characteristics through Wnt/β-catenin signaling. Environ Toxicol. (2023) 38:2894–903. doi: 10.1002/tox.23924, PMID: 37551626

[90] Sui Y Shi J Cai S Xiong T Xie B Sun Z. Metabolites of Procyanidins from *Litchi Chinensis* pericarp with xanthine oxidase inhibitory effect and antioxidant activity. Front Nutr. (2021) 8:676346. doi: 10.3389/fnut.2021.676346, PMID: 34621770 PMC8490629

[91] Wang K Chen X Chen Y Sheng S Huang Z. Grape seed procyanidins suppress the apoptosis and senescence of chondrocytes and ameliorates osteoarthritis via the DPP4-Sirt1 pathway. Food Funct. (2020) 11:10493–505. doi: 10.1039/d0fo01377c, PMID: 33175932

[92] Wang Z Li X Ren X Zhao S Chen W Fan C. Procyanidins extracted from the Lotus seedpod ameliorate cognitive impairment through CREB-BDNF pathway mediated LTP in APP/PS1 transgenic mice. Curr Pharm Biotechnol. (2023) 24:1560–7. doi: 10.2174/1389201024666230209142145, PMID: 36757040

[93] Yang B Sun Y Lv C Zhang W Chen Y. Procyanidins exhibits neuroprotective activities against cerebral ischemia reperfusion injury by inhibiting TLR4-NLRP3 inflammasome signal pathway. Psychopharmacology. (2020) 237:3283–93. doi: 10.1007/s00213-020-05610-z, PMID: 32729095

[94] Yin J Wang K Zhu X Lu G Jin D Qiu J. Procyanidin B2 suppresses hyperglycemia-induced renal mesangial cell dysfunction by modulating CAV-1-dependent signaling. Exp Ther Med. (2022) 24:496. doi: 10.3892/etm.2022.11423 35837062 PMC9257762

[95] Hao R Li Q Zhao J Li H Wang W Gao J. Effects of grape seed procyanidins on growth performance, immune function and antioxidant capacity in weaned piglets. Livest Sci. (2015) 178:237–42. doi: 10.1016/j.livsci.2015.06.004

[96] Wang W Xu M Diao H Long Q Gan F Mao Y. Effects of grape seed proanthocyanidin extract on cholesterol metabolism and antioxidant status in finishing pigs. Sci Rep. (2024) 14:21117. doi: 10.1038/s41598-024-72075-x, PMID: 39256553 PMC11387843

[97] Park JC Lee SH Park SK Hong JK Zhang ZF Cho JH. Effects of fruit by-product extracts supplementation on growth performance and nutrient digestibility in growing pigs. J Animal Sci Technol. (2013) 55:257–61. doi: 10.5187/JAST.2013.55.4.257

[98] Fang L Li M Zhao L Han S Li Y Xiong B. Dietary grape seed procyanidins suppressed weaning stress by improving antioxidant enzyme activity and mRNA expression in weanling piglets. J Anim Physiol Anim Nutr. (2020) 104:1178–85. doi: 10.1111/jpn.13335, PMID: 32189416

[99] Liu J Qiao Y Yu B Luo Y Huang Z Mao X. Functional characterization and toxicological study of Proanthocyanidins in weaned pigs. Toxins. (2023) 15:558. doi: 10.3390/toxins15090558, PMID: 37755984 PMC10535313

[100] Park JC Lee SH Hong JK Cho JH Kim IH Park SK. Effect of dietary supplementation of procyanidin on growth performance and immune response in pigs. Asian Australas J Anim Sci. (2014) 27:131–9. doi: 10.5713/ajas.2013.13359, PMID: 25049935 PMC4093277

[101] Li Y Feng Y Chen X He J Luo Y Yu B. Dietary short-term supplementation of grape seed proanthocyanidin extract improves pork quality and promotes skeletal muscle fiber type conversion in finishing pigs. Meat Sci. (2024) 210:109436. doi: 10.1016/j.meatsci.2024.109436, PMID: 38266434

[102] Xu M Chen X Huang Z Chen D Li M He J. Effects of dietary grape seed proanthocyanidin extract supplementation on meat quality, muscle fiber characteristics and antioxidant capacity of finishing pigs. Food Chem. (2022) 367:130781. doi: 10.1016/j.foodchem.2021.130781, PMID: 34391997

[103] Zhang Y Zhai Y Wei X Yang X Deng C Li Q. Effects of grape seed procyanidins on the lipid metabolism of growing-finishing pigs based on transcriptomics and metabolomics analyses. Meat Sci. (2024) 213:109504. doi: 10.1016/j.meatsci.2024.109504, PMID: 38555738

[104] Feng Y Chen X Chen D He J Zheng P Luo Y. Dietary grape seed proanthocyanidin extract supplementation improves antioxidant capacity and lipid metabolism in finishing pigs. Anim Biotechnol. (2023) 34:1–11. doi: 10.1080/10495398.2023.2252012, PMID: 37647084 PMC13353474

[105] O’Grady MN Carpenter R Lynch PB O’Brien NM Kerry JP. Addition of grape seed extract and bearberry to porcine diets: influence on quality attributes of raw and cooked pork. Meat Sci. (2008) 78:438–46. doi: 10.1016/j.meatsci.2007.07.011, PMID: 22062463

[106] Zhang HJ Jiang XR Mantovani G Lumbreras AEV Comi M Alborali G. Modulation of plasma antioxidant activity in weaned piglets by plant polyphenols. Ital J Anim Sci. (2014) 13:3242. doi: 10.4081/ijas.2014.3242

[107] Pistol GC Bulgaru CV Marin DE Oancea AG Taranu I. Dietary grape seed meal bioactive compounds alleviate epithelial dysfunctions and attenuates inflammation in Colon of DSS-treated piglets. Food Secur. (2021) 10:530. doi: 10.3390/foods10030530, PMID: 33806347 PMC7999447

[108] Fiesel A Gessner DK Most E Eder K. Effects of dietary polyphenol-rich plant products from grape or hop on pro-inflammatory gene expression in the intestine, nutrient digestibility and faecal microbiota of weaned pigs. BMC Vet Res. (2014) 10:196. doi: 10.1186/s12917-014-0196-5, PMID: 25323005 PMC4158062

[109] Pearce SC Mani V Boddicker RL Johnson JS Weber TE Ross JW. Heat stress reduces intestinal barrier integrity and favors intestinal glucose transport in growing pigs. PLoS One. (2013) 8:e70215. doi: 10.1371/journal.pone.0070215, PMID: 23936392 PMC3731365

[110] Hu CH Xiao K Luan ZS Song J. Early weaning increases intestinal permeability, alters expression of cytokine and tight junction proteins, and activates mitogen-activated protein kinases in pigs1. J Anim Sci. (2013) 91:1094–101. doi: 10.2527/jas.2012-5796, PMID: 23230104

[111] Li QH Yan HS Li HQ Gao JJ Hao RR. Effects of dietary supplementation with grape seed procyanidins on nutrient utilisation and gut function in weaned piglets. Animal. (2020) 14:491–8. doi: 10.1017/s1751731119002234, PMID: 31588892

[112] Wei X Li L Yan H Li Q Gao J Hao R. Grape seed procyanidins improve intestinal health by modulating gut microbiota and enhancing intestinal antioxidant capacity in weaned piglets. Livest Sci. (2022) 264:105066. doi: 10.1016/j.livsci.2022.105066

[113] Duarte ME Kim SW. Intestinal microbiota and its interaction to intestinal health in nursery pigs. Anim Nutr. (2022) 8:169–84. doi: 10.1016/j.aninu.2021.05.001, PMID: 34977387 PMC8683651

[114] Zhao T Shen X Dai C Cui L. Benefits of procyanidins on gut microbiota in Bama minipigs and implications in replacing antibiotics. J Vet Sci. (2018) 19:798–807. doi: 10.4142/jvs.2018.19.6.798, PMID: 30304891 PMC6265587

[115] Williams AR Krych L Fauzan Ahmad H Nejsum P Skovgaard K Nielsen DS. A polyphenol-enriched diet and Ascaris suum infection modulate mucosal immune responses and gut microbiota composition in pigs. PLoS One. (2017) 12:e0186546. doi: 10.1371/journal.pone.0186546, PMID: 29028844 PMC5640243

